# Supplementary material for: Molecular and Cellular Markers in Chlorhexidine-Induced Peritoneal Fibrosis in Mice
Source: Biomedicines. 2022 Oct 27;10(11):2726. doi: 10.3390/biomedicines10112726 (PMC9687430; doi:10.3390/biomedicines10112726)
Supplement: Supplementary file 1 [file biomedicines-10-02726-s001.zip › biomedicines-1944913-supplementary.pdf]

Table S1. Sequences of primers for qPCR.

Gene	Forward primer 5′ to 3′	Reverse primer 5′ to 3′
GAPDH	CAGTGGCAAAGTGGAGATT	CTTGACTGTGCCGTTGAA
TGFB1	TGGACACTCGATCGCTAC	AAGTGGGTGTTCTTAAATAGGG
COL1A1	TGAAGTCAGCTGCATACAC	AGTCCAGTTCTTCATTGCAT
ACTA2	GCTATGTGTGAAGAGGAAGAC	GCCCATTCCAACCATTACT
IL6	TCCAGTTGCCTTCTTGGGAC	GTACTCCAGAAGACCAGAGG
C6	TGGAATGCCAAAGGACCTCG	GCAACAACAAAGCCTCTGGG
C5	GAACAAACCTACGTCATTTCAGC	CGGGGACAAATTAACATAGCCTG
C3	CCAGCTCCCCATTAGCTCTG	GCACTTGCCTCTTTAGGAAGTC
C2	CAGGCTTGAACTTTGACCTTGG	GTAAGAGTAAGGCTGTCGGC
FN	CACTTACACCATCCAAGTCC	TCCCAGGAGACAGTAAGC
MMP3	AGGTGTTGACTCAAGGGTGG	TTGGGTCAAATTCCAACTGCG
MMP14	GCCCTCTGTCCCAGATAAGC	TTGGTTATTCCTCACCCGCC
CD55	ACCTCCACTCCCAGGAAAAG	TAGAGGAGACACCGACTAGCC
SAA1/2	GTAATTGGGGTCTTTGCC	TTCTGCTCCCTGCTCCTG
SAA3	TGCCATCATTCTTTGCATCTTGA	CCGTGAACTTCTGAACAGCCT
CTGF	CAAGCTGCCTGGGAAATG	ATGTGTCTTCCAGTCGGTAG
